# Evidence That Mutation Is Universally Biased towards AT in Bacteria

**DOI:** 10.1371/journal.pgen.1001115

**Published:** 2010-09-09

**Authors:** Ruth Hershberg, Dmitri A. Petrov

**Affiliations:** Department of Biology, Stanford University, Stanford, California, United States of America; University of Arizona, United States of America

## Abstract

Mutation is the engine that drives evolution and adaptation forward in that it generates the variation on which natural selection acts. Mutation is a random process that nevertheless occurs according to certain biases. Elucidating mutational biases and the way they vary across species and within genomes is crucial to understanding evolution and adaptation. Here we demonstrate that clonal pathogens that evolve under severely relaxed selection are uniquely suitable for studying mutational biases in bacteria. We estimate mutational patterns using sequence datasets from five such clonal pathogens belonging to four diverse bacterial clades that span most of the range of genomic nucleotide content. We demonstrate that across different types of sites and in all four clades mutation is consistently biased towards AT. This is true even in clades that have high genomic GC content. In all studied cases the mutational bias towards AT is primarily due to the high rate of C/G to T/A transitions. These results suggest that bacterial mutational biases are far less variable than previously thought. They further demonstrate that variation in nucleotide content cannot stem entirely from variation in mutational biases and that natural selection and/or a natural selection-like process such as biased gene conversion strongly affect nucleotide content.

## Author Summary

Natural selection sorts through the variability generated by mutation and biases evolution toward fitter outcomes. However, because mutation is itself not entirely random it can also bias the direction of evolution independently of selection. For instance, it is often assumed that the extreme variation observed in nucleotide content among bacteria (from ∼20% to ∼80% GC) is predominantly driven by extreme differences in mutational biases towards or away from GC. Here, we show that bacterial lineages that recently developed clonal, pathogenic lifestyles evolve under weak selection and that polymorphisms in these bacteria can be used as a fair proxy for mutational spectra. We analyze large sequence datasets from five clonal pathogens in four diverse bacterial clades spanning most of the range of genomic nucleotide content. We find that, surprisingly, mutation is AT-biased in every case to a very similar degree and in each case it is dominated by transitions from C/G to T/A. This demonstrates that mutational biases are far les variable than previously assumed and that variation in bacterial nucleotide content is not due entirely to mutational biases. Rather natural selection or a selection like process such as biased gene conversion strongly affect nucleotide content in bacteria.

## Introduction

Mutation generates the variability on which natural selection acts. Mutation is not an entirely stochastic process, as it acts according to certain deterministic biases. Because of this, biases in the outcome of the evolutionary process result not only from selection but also from the biases of mutation. In order to understand evolution it is therefore necessary to elucidate mutational biases and the ways in which these biases themselves change in evolution.

**Figure 1:**
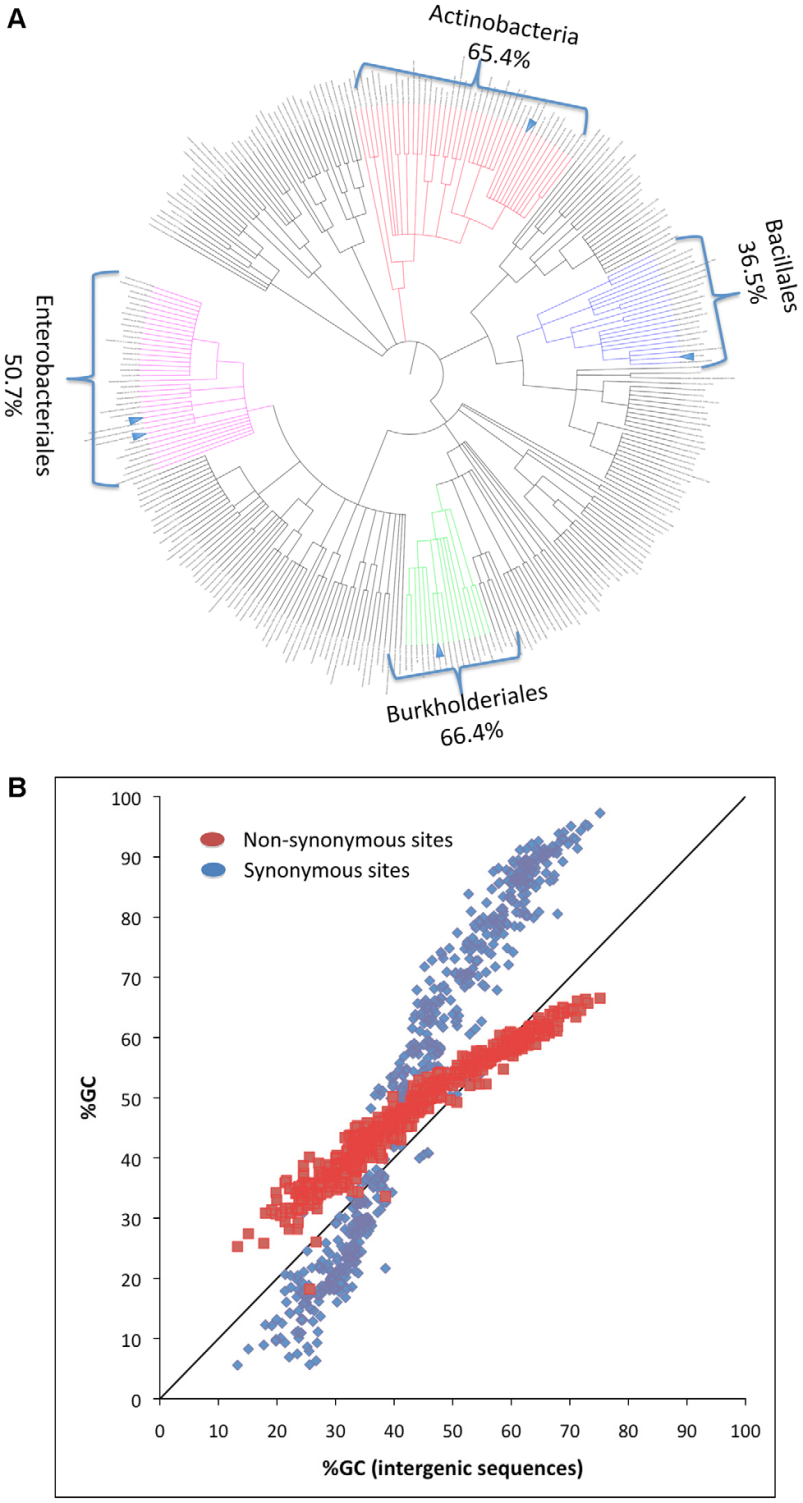
Phylogenetic and genomic variation in GC content. (A) The phylogeny of the four broad clades examined in this study. It was built using the iTOL webpage (http://itol.embl.de/). Average GC content of the different broad clades are indicated on the margins. Small blue triangles represent the five lineages of clonal pathogens used in the analysis. (B) Genomewide observed GC content of synonymous and non-synonymous sites correlate with the intergenic GC content across bacterial genomes.

Nucleotide content variation is much more pronounced in bacteria compared to multi-cellular eukaryotes [1]. GC contents in bacteria vary from less than 25% to over 75% [1]–[3]. Related bacteria even from relatively broad phylogenetic groupings tend to show similar genomic nucleotide content [3]. For example bacterial genomes from the order Bacillales tend to be GC-poor, from the order Enterobacteriales to have intermediate GC contents, and from the phylum Actinobacteria to be GC-rich (Figure 1A). In addition, GC content values measured at different functional site categories (intergenic, synonymous, and non-synonymous) show highly correlated patterns of variation across bacteria [4] (Figure 1B). These observations suggest that forces determining GC content in bacteria operate both genome-wide and consistently over long periods of time. One possibility is that the main force driving nucleotide content variation in bacteria is mutation. This possibility has often been assumed true (for example see [2], [4]–[7]). Under this assumption, clades that are GC rich are clades in which mutation has been consistently biased towards GC while clades that are AT rich are clades in which mutation has been consistently biased towards AT. If true, bacterial mutational biases must to be extremely variable to be able to generate the extreme variation observed in bacterial nucleotide content.

A second possibility is that it is not variation in mutational biases that leads to variation in nucleotide content, but rather variation in the relative probabilities of fixation of A/T to G/C and G/C to A/T mutations [1], [3], [8]. When considering changes to nucleotide content, differences in fixation probabilities can stem both from differences in the strength and direction of natural selection and differences in the rates of biased gene conversion (BGC) [1], [9], [10]. Natural selection affects the probability of fixation of an allele based on the alleles fitness advantage or disadvantage and the effective population size (N_e_) of the organism in question. Similarly BGC is also dependent on N_e_ and on the advantage or disadvantage the allele has. Here however, this advantage or disadvantage is not in fitness but rather in the increased or decreased probability of the allele to be passed on to the next generation through gene conversion [1], [10]. This increase or decrease is determined by recombination rates and by the conversion bias, which has been shown in many eukaryotes to be in favor of GC nucleotides compared to AT nucleotides [1], [9]. A recent study that showed that in *Escherichia coli* regions of low recombination tend to be more AT rich demonstrates that BGC may affect nucleotide content in bacteria in a similar manner [11].

In order to gain insight into mutational biases it is necessary to investigate the results of mutation in isolation from those of selection and BGC. When effective population sizes are small, the efficacy of both natural selection and BGC is severely reduced relative to stochastic processes and therefore sequence evolution is affected strongly by mutational biases. Mutation-accumulation experiments artificially reduce N_e_ of evolving laboratory cultures [12] and can thus be used to assess mutational biases in culturable bacteria. Similarly, reporter constructs have also been used to estimate mutational biases [13], [14]. However, without knowing the relative amount of time bacteria spend in different growth phases (logarithmic vs. stationary) and given that mutational rates and patterns vary between growth phases [15], [16] it could be difficult to estimate the true mutational biases operating in nature using such experimental approaches. An additional approach is to examine nucleotide substitutions at sites that are expected not to be subject to selection due to protein functionality, such as pseudogenes [17], or fourfold degenerate sites [18]. This approach is also problematic because while pseudogenes and fourfold degenerate sites are expected to be under no, or low selection for protein functionality, they should be subject to the same levels of selection on nucleotide content as the rest of the genome.

A good way to estimate mutational biases is to analyze the patterns of single nucleotide polymorphisms (SNPs) within species. Population genetic studies have shown that natural selection and other selection-like processes are less efficient in affecting patterns of nucleotide polymorphisms among very closely related strains compared to nucleotide differences between distantly related strains or species [19], [20]. Thus SNPs should better reflect the mutational patterns compared to substitutions between species. The analysis of SNPs has been used to investigate the mutational biases of a number of AT-rich eukaryotic genomes, such as Drosophila [21]–[23]. However, using this methodology in bacteria has been problematic due to a severe blurriness in species boundaries among prokaryotes [24]. As a result of such blurriness quite often strains sharing a “species” name (such as *E. coli*) are in fact quite diverged. While it is difficult to define species in bacteria, severely reduced selection has been observed among very closely related bacterial strains of some strictly clonal pathogens, such as strains belonging to the *Mycobacterium tuberculosis* cluster (MTBC) [25]. This can be explained by the fact that strains belonging to such lineages of pathogens are extremely closely related and thus these lineages may be good proxies of “species” and nucleotide differences between them can be viewed as polymorphisms. In addition, the lifestyle and clonality of such pathogens is likely to lead to small N_e_, further reducing efficacy of natural selection [25]. The patterns of SNPs among closely related strains of such clonal pathogens should thus reflect directly the predominant mutational biases.

Here we estimate mutational biases by analyzing SNPs extracted from large sequence datasets of five lineages of clonal pathogens (including MTBC) from four broad clades of bacteria that span virtually the whole bacterial phylogeny and the range of bacterial nucleotide contents (Figure 1A): Bacillales (AT rich), Enterobacteriales (intermediate GC), Actinobacteria (high GC), and Burkholderiales (high GC). We find that in all lineages mutation is biased towards AT, and that G/C to A/T transitions are always predominant. Previous studies indicate that mutation may be universally AT-biased in eukaryotes [21], [26]–[31]. Our results together with additional studies that have focused on Enterobacteriales (*E. coli*, *Shigella*, and *Salmonella typhimurium*) [32], [33] demonstrate that mutation may be universally AT-biased in bacteria as well. These findings contradict the long-held view that mutational biases are the main contributors to variation in bacterial nucleotide content and are therefore highly variable among bacteria. Rather they suggest that nucleotide content in bacteria is strongly affected by variation in the relative rates of fixation of AT to GC and GC to AT mutations and that mutational biases are far less variable than previously thought.

## Results

### Sequence data from lineages of clonal pathogens are extremely suited to the study of bacterial mutational biases

We focused on five lineages of clonal pathogens from four diverse bacterial clades (Table 1). The five lineages we investigated are unique in their suitability for this type of analysis because they provide us with sufficient amounts of available sequence data for sufficiently closely related strains in which we can demonstrate a genome-wide relaxation in the efficacy of natural selection. The chosen strains are indeed very closely related with each lineage exhibiting less than 0.5 pairwise differences per gene (Table 1). However, because of the availability of multiple whole genome sequences in each lineage the total number of SNPs is substantial (Table 1), ranging from 165 to 1877. In addition, these lineages are thought to be clonal [34]. Thus, horizontal gene transfer should occur only rarely, if at all and should not strongly influence our ability to infer the ancestral and derived states of mutations. Finally, the inference of SNPs from such closely related sequences is almost trivial, as alignment programs do much better when sequences are highly similar. Therefore, we expect to have no biases introduced through misalignment of sequences.

**Table 1:** Clonal pathogen lineages analyzed in study.

*Clonal pathogen*	*Source of SNPs*	*Outgroup*	*Max pairwise variability* a	*dN/dS*	*#SNPs*		
					Intergenic	Non-synonymous	Synonymous
*Bacillus anthracis*	Alignments of 18 fully and partially sequenced strains	*B. thuringiensis*	0.3	0.58	322	239	112
*Salmonella typhi*	SNPs provided by Holt et al. [52] based on the sequencing of 19 strains	None (phylogenetic tree)	0.4	0.45	260	961	656
*Yersinia pestis*	Alignments of 7 fully sequenced strains	*Y. pseudotuberculosis*	0.2	0.64	118	345	162
*Burkholderia mallei*	Alignments of 11 fully and partially sequenced strains	*B. thailandensis*	0.1	0.47	44	70	51
MTBC	Alignment of 89 genes sequenced in 107 strains	*M. canettii*	0.5	0.59	NA	226	136

a The average pairwise diversity per gene of the two most diverged strains within the lineage.

We assessed whether the patterns of SNPs in these data are indeed weakly affected by natural selection by estimating the ratio of non-synonymous and synonymous differences per non-synonymous and synonymous site (dN/dS) [35], [36] across all alignable proteins within each dataset (Materials and Methods). If selection is strong, dN/dS should be much smaller than 1 as it would efficiently remove most non-synonymous mutations [35], [36]. For example, comparisons of *E. coli* strains yields dN/dS values of approximately 0.05 [37]. In contrast, for MTBC where multiple lines of evidence suggest that natural selection is severely reduced [25], dN/dS goes up to 0.59. In our dataset, dN/dS values for the other four lineages range between 0.45 and 0.64 (Table 1, Materials and Methods). This suggests that selection is indeed relaxed in a genome-wide manner in these genomes and thus that the pattern of SNPs should be reflective of the mutational biases in these lineages.

Relaxed selection over long evolutionary timescales can lead to extreme genome reduction and the loss of many repair pathways, which could affect mutational patterns [1], [38]. The pathogens we use in this study have suffered only a short-term relaxation in the efficiency of selection and there is no indication that any of them have lost repair functions. To further substantiate that these pathogens are not likely to have suffered loss of repair functions we examined whether any of the repair genes annotated in close relatives of the examined pathogens that are not evolving under inefficient selection, have been lost in the pathogens. We found that in *B. anthracis*, *B. mallei*, *S. typhi* and *Y. pestis* there has been no loss of repair genes and that in all cases the repair genes are highly similar to those found in the outgroups (Materials and Methods, Table S1). In the case of MTBC, since the closely related outgroup strain *M. canettii* is not fully sequenced we could only compare the genes present in fully sequenced strains of MTBC to those present in a more distantly related outgroup, *M. marinum*. All but one of the repair genes found in *M. marinum* are also found in MTBC (Table S1). The *M. marinum* gene that is not found in MTBC is an un-named gene of unclear function. These results together with a lack of previous evidence for loss of repair functions in these well studied pathogens makes it unlikely that these pathogens have lost repair functions. It is even less likely that all of them suffered similar losses of repair functions.

### Mutation is AT-biased independently of the current nucleotide content of bacterial clades

**Figure 2:**
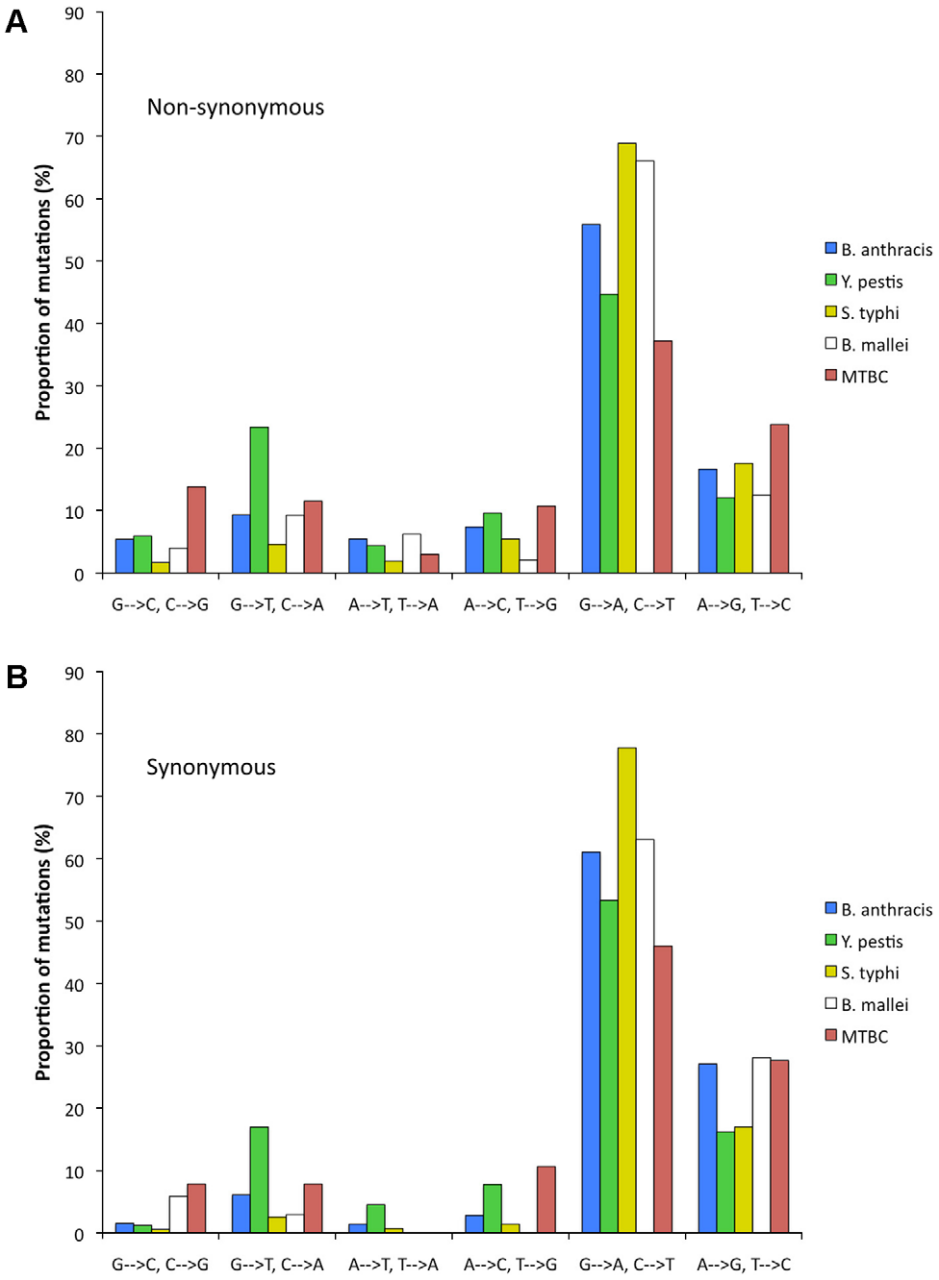
Relative rates of the six nucleotide pair mutations. The most common mutation is always G/C to A/T transitions. The rates are normalized for the unequal nucleotide content of the five different lineages (Materials and Methods). (A) non-synonymous SNPs. (B) synonymous SNPs.

We polarized the SNPs (Materials and Methods) and classified changes into six possible types of mutations (G/C to A/T, G/C to T/A, G/C to C/G, A/T to G/C, A/T to C/G, and A/T to T/A). The relative rate of each of the six mutation types was calculated after normalizing for the current GC content at the studied positions (Materials and Methods). For all five lineages, irrespective of current genomewide nucleotide content, the predominant mutation is G/C to A/T transition (Figure 2).

It is important to remember that relaxation of selection in the studied lineages is fairly recent and that nucleotide content is a slowly evolving trait. Therefore, if driven by selection or BGC, nucleotide content should not have had time to reach a new mutational equilibrium. However, if nucleotide content is driven predominantly by mutation, and selection and BGC do not strongly affect nucleotide content the genomic nucleotide contents should already be at the mutational equilibrium. We test whether nucleotide content is at equilibrium by comparing the number of GC→AT and AT→GC changes observed in each dataset. Under equilibrium these numbers will be equal. The results of such comparisons (Table 2) clearly show that the lineages with intermediate (*Salmonella typhi* & *Yersinia pestis*) and high (*Burkholderia mallei* & MTBC) nucleotide contents are currently far from equilibrium and that GC→AT changes are much more frequent. These results are statistically significant for all but the intergenic dataset of *B. mallei*, in which a small number of SNPs leads to very low statistical power (Table 2).

**Table 2:** Nucleotide contents of clonal pathogens with intermediate and high GC contents are far from equilibrium.

*Clonal pathogen*	*Current GC content*	*Sites*	*#GC→AT* a	*#AT→GC* a
MTBC	High	Synonymous	**103** (84, 123)	**18** (10, 26)
MTBC	High	Non- synonymous	**127** (105, 151)	**58** (43, 73)
B. mallei	High	Synonymous	**45** (33, 59)	**2** (0, 5)
B. mallei	High	Non-synonymous	**57** (42, 73)	**7** (2, 12)
B. mallei	High	Intergenic	27 (17, 38)	12 (6, 19)
Y. pestis	Intermediate	Synonymous	**116** (95, 138)	**37** (26, 48)
Y. pestis	Intermediate	Non-synonymous	**230** (201, 259)	**79** (63, 97)
Y. pestis	Intermediate	Intergenic	**69** (52, 85)	**36** (25, 47)
B. anthracis	Low	Synonymous	44 (32, 57)	64 (49, 79)
B. anthracis	Low	Non-synonymous	**136** (114, 158)	**75** (58, 93)
B. anthracis	Low	Intergenic	141 (119, 166)	151 (128, 175)
S. typhi	Intermediate	Synonymous	**570** (524, 619)	**79** (62, 97)
S. typhi	Intermediate	Non-synonymous	**707** (654, 760)	**220** (192, 248)
S. typhi	Intermediate	Intergenic	**189** (164, 215)	**65** (50, 81)

a 95% Confidence intervals appear in parenthesis. Numbers appear in bold if there is a statistically significant (p<0.05) difference between number of GC→AT and number of AT→GC changes.

**Figure 3:**
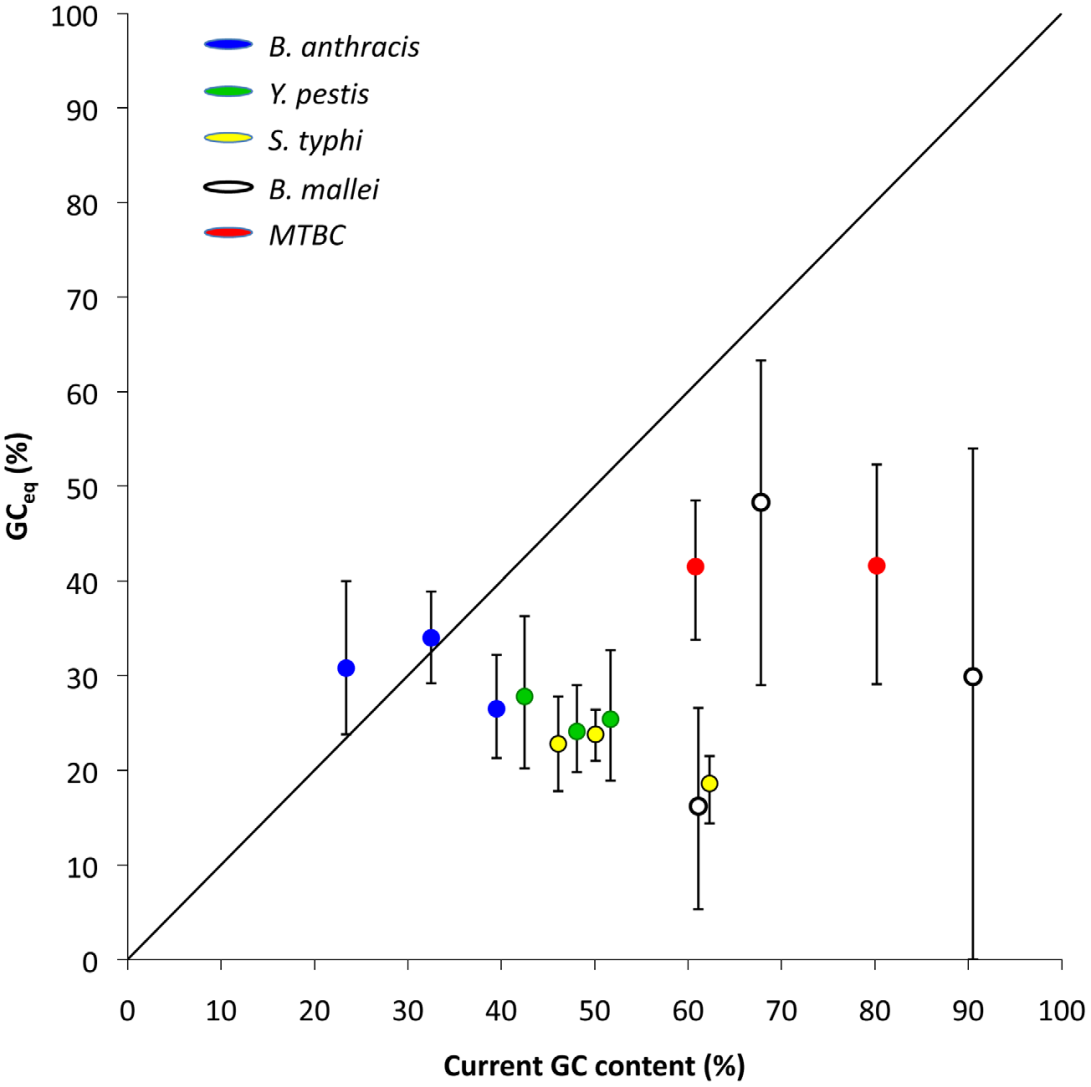
Equilibrium GC content (GC_eq_ ) and the observed GC content in the five studied clonal pathogen lineages. GC_eq_ is calculated from the estimated rates AT to GC and GC to AT mutations (r_AT→GC_ and r_GC→AT_) as 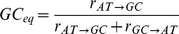. GC_eq_ values are significantly lower than the observed GC content at all site categories (intergenic, synonymous and non-synonymous) and for all four lineages of clonal pathogens with either intermediate or high GC contents. MTBC intergenic SNPs were not available for analysis. Error bars depict 95% confidence intervals for GC_eq_. No correlation is observed between GC_eq_ and current GC for the clonal pathogen lineages (*P*≤1).

The above results indicate that under continued relaxed selection the lineages with intermediate or high GC content will evolve to become more AT rich. It is easy to calculate the expected equilibrium GC content based on the mutational rates we found (GC_eq_, Materials and Methods) [1], [8], [27]. Such a calculation shows that for all lineages GC_eq_ is lower than 50% (Figure 3, Table 3). In other words, mutational biases by themselves should lead to AT-rich genomes. Furthermore, if nucleotide content is primarily determined by the mutational biases of the genome, GC_eq_ should approximate the observed GC content genomewide, However, for the four clonal pathogen lineages with either intermediate or high GC contents, GC_eq_ is always significantly lower than the current genomewide GC at all types of sites (Figure 3, Table 3). Finally, no significant correlation was observed between GC_eq_ and current GC across all site categories in all lineages (*r* = 0.09, Spearman correlation, n = 14, *P*≤1. Note that the 14 data points examined are not entirely independent. The calculated P-value should therefore be taken with a grain of salt, and is only provided to demonstrate that GC_eq_ does not appear to be correlated to current GC).

**Table 3:** Comparisons of current GC content and GC_eq_ for five clonal pathogen lineages.

*Clonal pathogen*	*Current GC intergenic*	*GC_eq_ intergenic* a	*Current GC non-synonymous*	*GC_eq_ non-synonymous* a	*Current GC synonymous*	*GC_eq_ synonymous* a
*Bacillus anthracis*	32.5	34(29.2, 38.9)	39.5	**26.5**(21.3, 32.2)	23.4	**30.8**(23.8, 40)
*Salmonella typhi*	46.1	**22.8**(17.8. 27.8)	50.1	**23.8**(21.2, 26.6)	62.3	**18.6**(15.7, 22.8)
*Yersinia pestis*	42.5	**27.8**(20.2, 36.3)	48.1	**24.1**(19.8, 29)	51.7	**25.4**(18.9, 32.7)
*Burkholderia mallei*	67.8	**48.3**(29, 63.3)	61.1	**16.2**(5.3, 26.6)	90.5	**29.9**(0, 54)
MTBC	62.8	Missing data	60.8	**41.5**(33.8, 48.5)	80.2	**41.6**(29.1, 52.3)

a 95% Confidence intervals appear in parenthesis. Bold font indicates GC_eq_ values are significantly different from corresponding current GC contents.

In order to show that these results are not an artifact of sequencing errors, we recalculated GC_eq_ after removing all cases in which the derived allele appears only in a single genome (singletons). While this reduces the number of SNPs per dataset and increases the error of the estimate, GC_eq_ values remain lower than the current GC for all datasets from lineages with intermediate or high GC contents and the results remain statistically significant in all the datasets except for the *B. mallei* intergenic sites (Table S2). These results together show that in five lineages of clonal pathogens, belonging to four broad clades that span a large portion of bacterial phylogeny (Figure 1A) mutation is consistently biased towards AT. Furthermore, these results suggest that in bacteria that are GC rich or have intermediate GC contents, it is an elevated fixation of GC-enriching mutations rather than a change in mutation bias that drives the elevated GC content.

**Figure 4:**
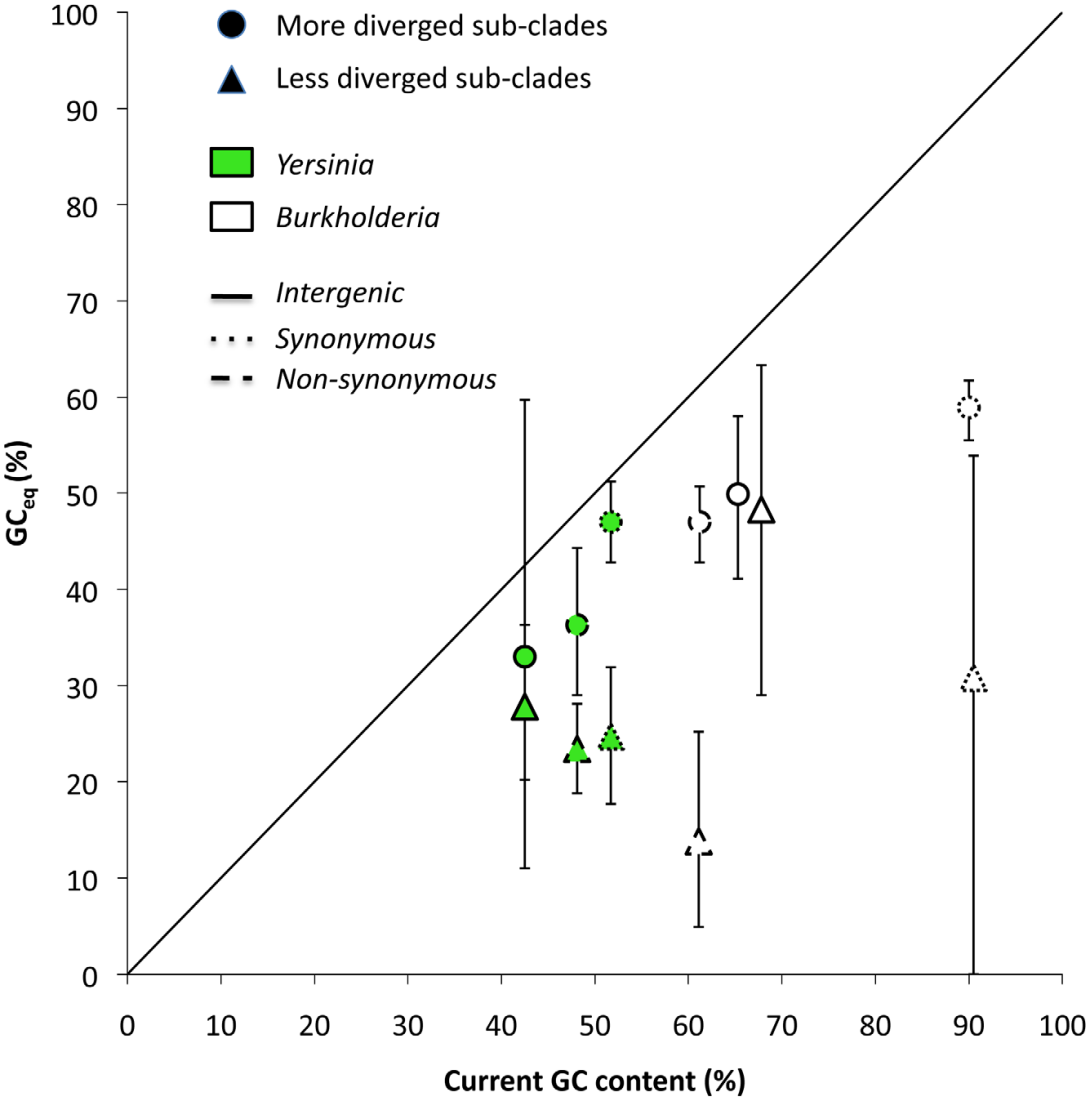
Comparison between GC_eq_ calculated using data from clonal pathogens and GC_eq_ calculated using data from more diverged lineages. GC_eq_ estimated using more diverged lineages is always more similar to and significantly correlated with current GC content values (*P* = 0.03).

Differences between genomes that are more distantly related to each other should reflect the effects of natural selection and/or BGC better. The GC_eq_ calculated based on such differences should be more similar to the observed GC content than that calculated based on SNPs from more closely related strains. To examine these predictions we analyzed two additional datasets in which sequences are still closely related enough to create reliable multiple sequence alignments for a relatively large number of their genes and intergenic regions, yet show higher divergence than the five lineages in Table 1 (Table 4). The first dataset was created by aligning sequences from *Y. pestis* and *Yersinia pseudotuberculosis* and examining the differences between these two lineages. The second dataset was created by aligning sequences from 10 strains of *Burkholderia pseudomallei*. These two datasets were selected because they can be naturally paired to two of the five datasets in Table 1 (*B. mallei* and *Y. pestis*). dN/dS values for these comparisons are lower than in the other five datasets (χ^2^ test, *P*<0.00001, Table 4), suggesting that the effects of selection are indeed more evident in these data. As predicted, the GC_eq_ values derived from differences between *Y. pestis* and *Y. pseudotuberculosis* and from *B. pseudomallei* comparisons are more similar to the current GC content at all types of sites compared to GC_eq_ calculated from the paired datasets of clonal pathogens (Figure 4). These results are statistically significant for the non-synonymous and synonymous site comparisons (Figure 4, Table 4). For intergenic comparisons a small number of identified *B. mallei* SNPs, and *Y. pestis*/*Y. pseudotuberculosis* differences makes it difficult to demonstrate statistical significance. Furthermore, the GC_eq_ values calculated based on the more diverged datasets are significantly correlated with the current genomewide GC content in *Y.pestis* and *B. pseudomallei* (*r* = 0.99, Spearman correlation, n = 6, *P*≤0.03). It is important to note that the GC_eq_ values derived from differences between *Y. pestis* and *Y. pseudotuberculosis* and from *B. pseudomallei* are still lower than current GC content for all comparisons (Figure 4). This indicates that even a relatively slight reduction in selection can lead to a certain reduction in GC content. A similar result was recently found for *E. coli* and *Shigella*, where a relatively slight reduction in the efficiency of selection [37] was shown to lead to an excess of GC→AT changes in *Shigella* compared to *E. coli* [32].

**Table 4:** Summary of results for two more distantly related lineages.

*Dataset*	*Max pairwise variability* a	*dN/dS*	*Current GC intergenic*	*GC_eq_ intergenic* b	*Current GC non-synnonymous*	*GC_eq_ non-synonymous* b	*Current GC synonymous*	*GC_eq_ synonymous* b
*Y.pestis*/*pseudotuberculosis*	4.9	0.1	42.5	33(11, 59.7)	48.1	**36.3**(29, 44.3)	51.7	**47**(42.8, 51.2)
*B. pseudomallei*	3.3	0.13	65.3	**49.9**(41.1, 58)	61.2	**47**(42.8, 50.7)	90	**58.9**(55.5, 61.7)

a The average pairwise diversity per gene of the two most diverged strains within the lineage.

b 95% Confidence intervals appear in parenthesis. Bold font indicates GC_eq_ values are significantly different from corresponding current GC contents.

### Nucleotide content of obligate intracellular bacteria matches those predicted based on estimates of mutational biases

Obligate intracellular bacteria are known to be evolving under extremely prolonged relaxed selection [38]. It is therefore possible that in these organisms nucleotide content will be strongly affected by mutation. The genomes of obligatory intracellular bacteria tend to be AT rich [38], indicating that mutation may be biased towards AT in these organisms. However, it is unknown whether the mutational biases of obligate intracellular bacteria reflect those of their clade members that are not living this lifestyle, since these organisms lack many of the repair genes which are present in their other clade members that are not evolving under such relaxed selection [38]. It is unclear how the absence of repair pathways affects the mutational biases of these bacteria. Lind and Anderson [33] have carried out mutation accumulation experiments in *Salmonella typhimurium* strains lacking the major DNA repair systems involved in repairing common spontaneous mutations caused by deaminated and oxidized DNA bases. They found that in such strains of *S. typhimurium* mutation is strongly AT-biased but that unlike what we find for our pathogens, when these repair pathways are absent transversions become much more frequent than transitions [33]. The question however remains of whether loss of repair pathways changes the AT bias of mutation in obligate intracellular bacteria.

It is also unclear whether the nucleotide contents of obligate intracellular bacteria are currently at mutational equilibrium. Even though these organisms have been subject to prolonged, severe relaxations of selection it is possible that they have not yet reached nucleotide content equilibrium. It is also theoretically possible that the strength of selection in favor of GC nucleotides increases as genomes become more AT rich (a from of synergistic epistasis [26], [39], [40]). If this is the case it is possible that nucleotide content in obligate intracellular bacteria may be at a new selection-mutation equilibrium.

The questions of whether the mutational biases of obligate intracellular bacteria resemble those of their other clade members and of whether their nucleotide content is at mutational equilibrium can be addressed by comparing our estimates of GC_eq_ to the GC content of obligatory intracellular bacteria from the same clades. Among the four analyzed clades, two (Enterobacteriales, and Actinobacteria) include sequenced obligatory intracellular bacteria. Intriguingly, the GC contents of the obligate intracellular bacteria within these two clades are similar to the values of GC_eq_ which we calculated using clonal pathogen SNP data from their corresponding clades: GC_eq_ values calculated based on SNPs from the Enterobacteriales *S. typhi*, and *Y. pestis* were ∼22%, and ∼25% respectively. While other members of the order Enterobacteriales have a current GC content of 43–57%, obligate intracellular bacteria within this order have GC contents of 23–30%. Within the phylum Actinobacteria all genomes have GC content of over 50% except for the only sequenced obligate intracellular bacteria, *Tropheryma whipplei* that has a GC content of 46%. This is very close to the GC_eq_ calculated for the Actinobacteria MTBC (∼42%). These results suggest that the GC content of obligate intracellular bacteria corresponds to what we would expect to find in their non-obligate intracellular clade members at equilibrium if nucleotide content had been determined by mutation alone. This anecdotal evidence suggests that even though obligate intracellular bacteria do tend to lose many repair functions the extent of their mutational AT bias resembles that of their other clade members. Additionally, it appears that nucleotide contents of these bacteria are close to mutational equilibrium. The observation that obligate intracellular bacteria across additional clades tend to have high AT contents relative to their non-obligate intracellular clade members, and to have GC contents lower than 50% (Table S3, Figure S1) may therefore support the generality of AT-biased mutation in bacteria beyond the four phylogenetically diverse broad clades examined in this study.

## Discussion

Differences in the patterns of mutation have been assumed to be the most likely explanation for the vast variation observed in nucleotide content across bacteria (for example see [2], [4]–[7]). Given that bacterial nucleotide content varies between >75% GC and >75% AT, this assumption implies that point mutation biases are extremely variable among bacteria. Our results demonstrate that mutational biases are in fact very similar across bacteria. Mutation appears to be dominated by C/G to T/A transitions and is AT-biased in every studied instance, even in bacteria with high genomic GC contents. At the same time, it is important to note that mutational biases need not be entirely constant in different bacteria. In fact, our results demonstrate that in the Actinobacteria mutation appears to be less strongly biased towards AT than in the other clades examined.

Due to a severe relaxation in natural selection, the recent evolution of the clonal pathogens investigated in this study should be predominantly driven by mutation. However, it is important to note that natural selection is not entirely absent in these pathogens and that mutations of severe deleterious effect will still be removed by selection. In MTBC we have evidence that selection is relaxed to the point that it does not distinguish between mutations that alter amino acids that are conserved across all Mycobacteria and are presumed to be under strong constraint and those that are variable in Mycobacteria and are likely to be under much weaker constraint [25], indicating that only extremely strongly deleterious mutations are being purged by selection. Yet it is still possible that our estimates of mutational biases are somewhat affected by the residual effects of natural selection on strongly deleterious mutations. If such residual effects of selection affect our results they are likely to make our estimates of the extent of the AT bias somewhat conservative. This is demonstrated by our finding that within a given clade a stronger bias towards AT is observed in clonal pathogens where selection is severely relaxed compared to closely related lineages subject to more efficient selection. This being said, our observation that within a broad clade obligate intracellular bacteria evolve to a GC content that matches very closely the one predicted based on our estimates of mutation suggests that our estimates of the extent of AT bias are quite reliable.

In an additional study, which is published back-to-back with our study in this issue of PLoS Genetics, Hildebrand *et al.* investigated mutational patterns by examining changes in fourfold degenerate codons in a large number of bacterial lineages with divergence under 10% at these sites [41] and argue for the AT bias of mutation in GC rich bacteria. The argument of Hildebrand *et al.* is complicated by two factors. First, natural selection in such lineages can remain strong as exemplified by *E. coli* [37] and *B. pseudomallei* (studied here) and much stronger than in the five clonal pathogen strains studied here. Second, the inference of mutational patterns from fourfold degenerate sites alone is complicated by a strong correlation of the GC content of selectively favored codons at the level of translation and the GC content of genomes [42], making it possible that some of the detected effects are related to natural selection at the level of translation. Both of these factors imply that the mutational patterns inferred by Hildebrand *et al.* are much less precise than those presented here. Nevertheless, the totality of the evidence in Hildebrand *et al.* is consistent with the generality of AT-biased mutation across bacteria, especially given their focus on a much larger number of bacterial lineages than presented here.

A prolonged severe relaxation of selection in obligate intracellular bacteria has led to massive loss of repair genes in these bacteria [38]. In the clonal pathogens studied here relaxation of selection occurred much more recently and there is no evidence for loss of repair functions in these genomes. In fact, we show that none of these pathogens have lost any of the repair genes encoded by their closely related outgroups that are evolving under efficient selection. Additionally, a previous study that investigated the pattern of mutation in strains of *S. typhimurium* with deficient repair functions has shown that in such strains transversions become much more frequent than transitions [33]. In contrast, in all of the five pathogens we examined mutation was strongly biased towards transitions, rather than transversions. We cannot show conclusively that no repair functions have been lost in any of these five pathogens. However, we believe that the lack of evidence for any such loss, the consistency of the mutational biases observed across all five datasets examined, and the bias towards transitions rather than transversions, make it reasonable to assume that our results are unaffected by a significant loss in repair functions.

We show that within broad clades obligate intracellular bacteria evolve to a GC content that matches very closely the GC content predicted at equilibrium based on our estimates of mutation rates of clonal pathogens belonging to the same clade. This together with the long-standing observation that the vast majority of obligate intracellular bacteria tend to have extremely AT rich genomes [38] supports the generality of AT-biased mutation in bacteria. It is however important to note the recent identification of an obligate intracellular bacterium, *Candidatus Hodgkinia cicadicola* that has a GC-rich genome (58.4% GC) [43]. It is possible that this bacterium constitutes an exception to the universality of AT-biased mutation and that mutation in this organism is GC biased. However, it is also possible that this is not the case and that this bacterium is GC-rich for other reasons (such as due to natural selection or BGC). Further studies will be needed to determine whether *Candidatus Hodgkinia cicadicola* indeed has exceptional mutational biases.

By demonstrating that variation in nucleotide content in bacteria is not generally driven by differences in mutational biases we demonstrate that natural selection or another selection-like process such as BGC must play a dominant role in nucleotide content variation, particularly in driving intermediate to high GC content in many bacterial lineages. At this point it is unclear how much of this variation is driven by selection and how much by BGC.

If natural selection plays a strong role in determining GC content it suggests that in many bacteria there are no truly neutrally evolving sites. The nature of such selection remains obscure. Because GC content correlates strongly across coding and noncoding sites genome-wide, natural selection acting on GC content probably relates to genome-wide functions such as replication or DNA maintenance and is less likely to be related to gene expression. Previous studies attempted to associate GC content with environmental factors such as growth temperature [44], [45], exposure to UV [8], oxygen requirements [46], and the ability to fix nitrogen [47]. While these studies were thought by some to be inconclusive [3], [44], [48]–[51] they provide the best current explanations for the possible involvement of selection in determining nucleotide content. However, considering that bacteria belonging to such broad clades as Actinobacteria have similar genomic nucleotide contents even though they are exposed to different environments it becomes tempting to speculate that environmental variables may not be the only underlying determinants for the natural selection acting on nucleotide content. It is possible that selection on nucleotide content is also driven by more intra-organismal factors that can affect entire clades irrespective of environment. Examples of such factors can be the ability of the replication machinery to work better on GC or AT rich sequences, DNA packaging, defense against phages or creating barriers for horizontal gene transfer. More studies need to be carried out to probe the possible involvement of selection in determining bacterial nucleotide content.

In order for an increase in BGC to explain GC richness, recombination should be pervasive enough in GC rich bacteria to drive GC contents that are elevated substantially above those observed even in sexually reproducing eukaryotes. It is very likely that per generation advantage given to GC nucleotides through gene conversion (which is determined by recombination rates and by the conversion bias [1], [10]) is significantly higher in sexually reproducing eukaryotes than in prokaryotes for which recombination is assumed to be less frequent [1]. However, it is possible that BGC may still affect some bacteria more strongly than eukaryotes, if N_e_ is increased in these bacteria by a higher factor than the factor by which the advantage given to GC nucleotides through gene conversion is decreased. This is an intriguing possibility, opening up new avenues of research into recombination rates and variability in N_e_ among bacteria.

It is possible to use the mutational rates we calculated to estimate the strength with which selection, and/or BGC were acting on nucleotide content in the examined clonal pathogens prior to their recent relaxations of selection. Such calculations (Table S4) show that such selection is always weak (s≤1/N_e_), which would be expected considering GC content is always intermediate. This demonstrates that the selective or BGC advantage of GC over AT nucleotides need not be high in order to explain the vast variation in GC contents observed in bacteria. However, it is important to note that such calculations make a number of assumptions that may not be reasonable. One such assumption is that selection acts uniformly across sites and that there is no synergistic epistasis (i.e. that the intensity of selection does not change with changes in nucleotide content). An additional assumption is that there are no competing selective forces. This second assumption is clearly incorrect when it comes to non-synonymous sites. Even if selection on nucleotide content in these sites were strong enough to drive them to use only GC nucleotides it is highly likely that selection for protein function would not allow this to happen. It is therefore unclear whether selection on nucleotide content is in fact always weak.

In this study we demonstrate the great utility of clonal pathogens for the study of mutational biases in bacteria. We investigated five such clonal pathogens from four very diverse clades. We showed that in every studied case and across all site categories point mutation is consistently biased towards AT and that the most frequent mutations are always G/C→A/T transitions, thus demonstrating that the biases of point mutation are much less variable than was previously assumed. By identifying additional bacteria evolving under strongly relaxed selection and conducting deep sequencing of such bacteria it should be possible to address additional questions regarding mutational patterns of bacteria, including the variability in the rates of insertions and deletions across bacteria and mutation clustering along bacterial chromosomes.

Recent studies have shown that mutation appears to be universally AT-biased in eukaryotes [21], [26]–[31]. Our results demonstrating that this may also be the case in prokaryotes therefore show that mutation may in fact be AT-biased in all living organisms (although it is important to note that we do not yet have good estimates of the mutational biases of Archea). Not only is mutation AT-biased in all instances studied, but the specific pattern of mutation is always consistent. The most common mutations are always G/C to A/T transitions. These results make it tempting to speculate that the predominant mutations are simply the result of the lability of cytosine to deamination, and that this pattern shows through despite possible variability in DNA replication and repair mechanisms [27].

### Concluding remarks

In this study we used data from five strictly clonal pathogens to analyze the variation in point mutation biases in bacteria. These pathogens are uniquely suitable for such analyses as they can be shown to be evolving under selection that is severely inefficient relative to stochastic processes. Unlike obligate intracellular bacteria that have been evolving under inefficient selection for long evolutionary times and have lost much of their repair pathways these clonal pathogens have experienced only a short-term relaxation in selection efficiency and are likely to have intact repair mechanisms. Their mutational biases should therefore reflect those of their other clade members that are not subject to inefficient selection. We demonstrated that even though these five pathogens belong to four very diverse clades with very different nucleotide contents mutation in all of them is biased towards AT, and that the most frequent mutations are always G/C to A/T transitions. Our results show that variation in nucleotide content in bacteria cannot be explained by variation in mutational biases and that biases in point-mutation appear to be far less diverse among bacteria than was previously assumed.

## Materials and Methods

### Data sources

*Salmonella typhi* SNPs were taken from the study of Holt et al [52].

MTBC sequences of 89 genes from 107 MTBC strains and one outgroup strain (*M. canettii*) were taken from our previous study [25].

18 fully and partially sequenced genomes of *B. anthracis*, 11 of *B. mallei* and 10 of *B. pseudomallei* were taken from the Pathema database [53], together with the completed sequences of the outgroup strains *Bacillus thuringiensis* and *Burkholderia thailandensis*.

Seven fully sequenced strains of *Y. pestis*, four fully sequenced strains of *Y. pseudotuberculosis* and the fully sequenced outgroup strain *Y. enterocolitica* were downloaded from the NCBI FTP server (ftp.ncbi.nih.gov).

### SNP extraction and polarization

Within each dataset one strain was randomly selected to be the reference genome. The protein sequences of the reference genome were compared using the FASTA algorithm [54] to the protein sequences of all the other strains within the dataset including the outgroup strain. In such a way orthologs were identified as the best reciprocal hits. To prevent false identification of orthologs conservation of gene order along the chromosome was also required. More specifically if in one genome gene X is adjacent to genes Y and Z along the chromosome, and in another genome gene X′ is adjacent to genes Y′ and Z′ and if the best reciprocal hit of gene X in the other genome is gene X′, gene X′ will be considered the ortholog of gene X only if genes Y′ and Z′ are also gene Y and Z's reciprocal best hits.

Multiple sequence alignments (MSAs) were created at the protein level for genes for which orthologs could be identified in all of the strains within the dataset and in the outgroup strain. The MSAs were created using the clustalW alignment program. DNA/codon level MSAs were then created based on the protein level alignments by threading the DNA sequences unto the protein alignment. SNPs were extracted from these MSAs and the identities of the ancestral and derived alleles (polarization) were determined according to the outgroup strain sequence. To prevent false identification of SNPs due to misalignments we excluded SNPs from genes with more than 10 SNPs from further analyses.

In a similar manner the intergenic regions of the reference genome were compared at the DNA level to the intergenic regions of all other strains and of the outgroup. Intergenic sequences were considered orthologous if they were reciprocal best hits and if they could be aligned across their entire sequence. MSAs were created at the DNA level for intergenic sequences for which orthologs could be identified in all strains. To prevent false identification of SNPs due to misalignments (a problem that seemed to affect intergenic regions in particular) we excluded SNPs from intergenic sequences with more than 10 SNPs from further analyses. In the cases of *B. anthracis* and *B. mallei* this left us with very few SNPs when an outgroup strain was used. Therefore in these two datasets the intergenic SNPs were not polarized using the outgroup. Instead we assumed that the most frequent allele within the SNP is the ancestral allele, while the less frequent one is derived. An outgroup strain was used to polarize the intergenic SNPs in the remaining datasets.

### Calculating the relative rates of the six nucleotide pair mutations

In order to account for the unequal nucleotide content of the five different lineages we normalized the counts of the mutations from A/T to G/C, C/G, or T/A by multiplying them by 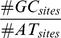, where #GC_sites_ and #AT_sites_ are the current genome wide number of GC or AT sites at the considered site category. In this way we determine the expected number of such mutations under equal GC and AT contents. In order to calculate the relative rates of each possible pairwise mutation each of the resulting counts (unaltered in the case of mutations from G/C, and normalized in the case of mutations from A/T) was multiplied by 100 and divided by the sum of these counts.

### Calculating GC_eq_

From the polarized SNPs calculating the number GC→AT and AT→GC changes (#GC→AT and #AT→GC) is straightforward. The rates of the two types of changes were calculated separately for intergenic, synonymous and non-synonymous sites as:
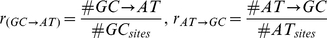
Where #GC_sites_ and #AT_sites_ are the current genome wide number of GC or AT sites at this site category in a randomly selected strain of the considered lineage. In order to calculate the current genome wide number of GC and AT sites for non-synonymous and synonymous sites for each genome we classified sites into synonymous and non-synonymous based on the method suggested by Nei and Gojobori [35]. According to this method sites will be considered entirely synonymous if no changes in them can lead to an amino acid change and will be considered entirely non-synonymous if all changes in them will cause an amino acid change. For sites in which some changes may change the amino acid while others will not the site is considered partially synonymous and partially non-synonymous according to the proportion of the changes that will lead to an amino acid change. We added to the relative GC or AT count of the sites category the proportion of the site which is attributable to that category. For example, if a site is 1/3 synonymous and 2/3 non-synonymous and the current base in this site is a C we added 1/3 of a count to #GC_sites_ of the synonymous sites and 2/3 of a count to #GC_sites_ of the non-synonymous sites. The GC contents we calculated for non-synonymous and synonymous sites were also used to draw Figure 1B.

Next, the expected equilibrium GC content based on these mutational rates (GC_eq_) was calculated as [1], [8]:
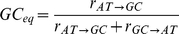



### Calculating confidence intervals of #GC→AT, #AT→GC and GC_eq_

1000 values were sampled from the Poisson distribution once with a mean of #GC→AT and once with a mean #AT→GC. This was done using the R program, rpois. The resulting values were sorted and used to estimate 95% confidence intervals for #GC→AT and #AT→GC. They were also used to recalculate GC_eq_ 1000 times and the resulting GC_eq_ values were sorted and used to estimate the 95% confidence intervals for GC_eq_.

### dN/dS calculations

dN/dS calculations were performed using the method of Nei and Gojobori [35]. dN/dS estimates were calculated for the entire genome rather than per gene. If in all considered genes we found ns non-synonymous SNPs and s synonymous SNPs and in the genome there are N nonsynonymous sites and S synonymous sites:
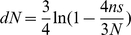


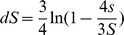



### Analysis of conservation of repair genes

Repair genes were identified based on genome annotation for five close relatives of the five pathogens, for which selection is efficient. Genes annotated as putative or hypothetical were excluded. For *Y. pestis* the close relative used was *Y. pseudotuberculosis*. For *B. mallei* the close relative used was *B. pseudomallei*. For *S. typhi* the close relative used was *S. typhimurium*, for *B. anthracis* the close relative used was *B. thuringiensis*. For MTBC this analysis was problematic as *M. canettii* which was used as an outgroup for MTBC strains in this study is not yet fully sequenced. We were therefore forced to use a more distantly related outgroup, *M. marinum*, which is far more diverged from MTBC than the other outgroups are from their pathogens. The orthologs of the repair genes from the outgroups were identified in the pathogens and the sequences of the pathogen versions of the genes were compared to those of the outgroups. The results of this analysis are summarized in Table S1.

## Supporting Information

Figure S1 GC contents of obligatory intra-cellular bacteria tend to be lower than those of other members of the same broad clades.
(3.00 MB TIF)

Table S1 Conservation of repair proteins in examined pathogens.
(0.09 MB DOC)

Table S2 Summary of results for five low-diversity sub-clades, with singletons removed.
(0.03 MB DOC)

Table S3 GC contents of obligate intracellular and non-obligate intracellular bacteria by cladea.
(0.07 MB XLS)

Table S4 Strength of selection or selection like processes for GC over AT nucleotides assuming constant selection and no competing selective pressures.
(0.04 MB DOC)
