# Supplementary figures and images for: Evidence That Mutation Is Universally Biased towards AT in Bacteria
Source: PLoS Genet. 2010 Sep 9;6(9):e1001115. doi: 10.1371/journal.pgen.1001115 (PMC2936535; doi:10.1371/journal.pgen.1001115)

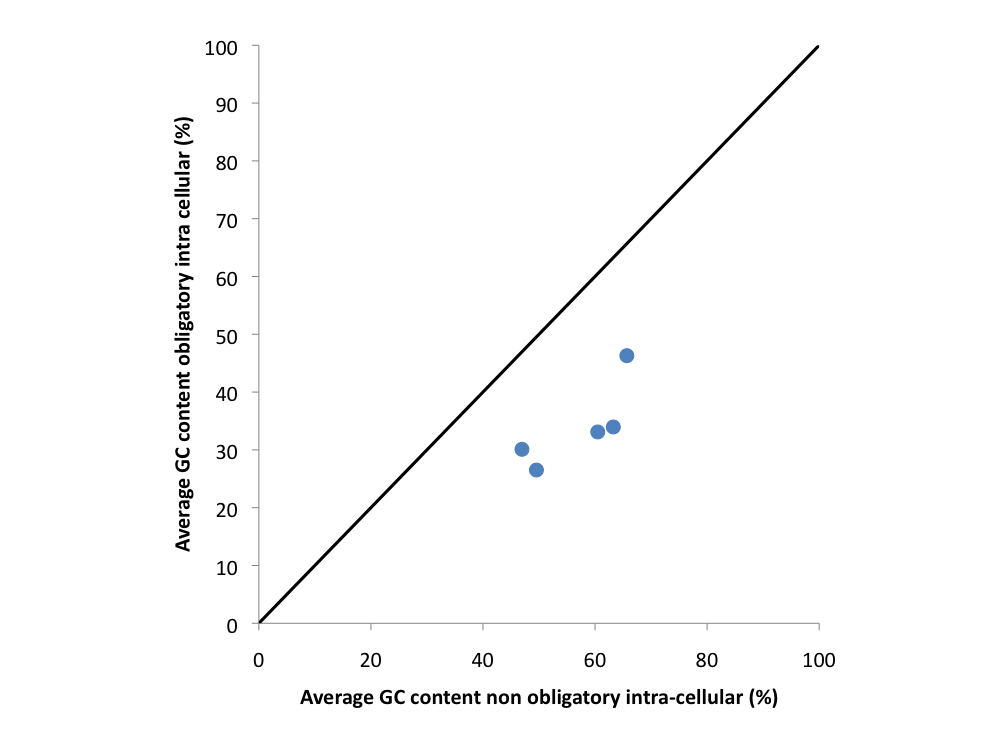

Supplement: Figure S1 — GC contents of obligatory intra-cellular bacteria tend to be lower than those of other members of the same broad clades. (3.00 MB TIF) [file pgen.1001115.s001.tif]
