# Supplementary material for: Evidence That Mutation Is Universally Biased towards AT in Bacteria
Source: PLoS Genet. 2010 Sep 9;6(9):e1001115. doi: 10.1371/journal.pgen.1001115 (PMC2936535; doi:10.1371/journal.pgen.1001115)
Supplement: Table S1 — Conservation of repair proteins in examined pathogens. (0.09 MB DOC) [file pgen.1001115.s002.doc]

Table S1. Conservation of repair proteins in examined pathogens

| Pathogen | Outgroup | Repair protein | % identity of repair protein between pathogen and outgroup |
| --- | --- | --- | --- |
| *Y. pestis* | *Y. pseudotuberculosis* | *RadC* | *100%* |
| *Y. pestis* | *Y. pseudotuberculosis* | *Bifunctional regulatory protein/DNA repair protein* | *98.6%* |
| *Y. pestis* | *Y. pseudotuberculosis* | *MutL* | *100%* |
| *Y. pestis* | *Y. pseudotuberculosis* | *Iron-sulfur cluster repair di-iron protein* | *99.6%* |
| *Y. pestis* | *Y. pseudotuberculosis* | *RadA* | *100%* |
| *Y. pestis* | *Y. pseudotuberculosis* | *MutS* | *99.9%* |
| *Y. pestis* | *Y. pseudotuberculosis* | *RecN* | *99.8%* |
| *Y. pestis* | *Y. pseudotuberculosis* | *Mfd* | *99.7%* |
| *Y. pestis* | *Y. pseudotuberculosis* | *RecO* | *100%* |
| *Y. pestis* | *Y. pseudotuberculosis* | *MutH* | *99.6%* |
| *Y. pestis* | *Y. pseudotuberculosis* | *MutY* | *99.5%* |
| *Y. pestis* | *Y. pseudotuberculosis* | *MutM* | *99.6%* |
| *Y. pestis* | *Y. pseudotuberculosis* | *Ung* | *100%* |
| *B. Mallei* | *B. Pseudomallei* | *RadC* | *100%* |
| *B. Mallei* | *B. Pseudomallei* | *RadA* | *100%* |
| *B. Mallei* | *B. Pseudomallei* | *Mfd* | *99.9%* |
| *B. Mallei* | *B. Pseudomallei* | *Base excision DNA repair protein* | *100%* |
| *B. Mallei* | *B. Pseudomallei* | *MutS* | *99.9%* |
| *B. Mallei* | *B. Pseudomallei* | *RecO* | *99.6%* |
| *B. Mallei* | *B. Pseudomallei* | *MutL* | *100%* |
| *B. Mallei* | *B. Pseudomallei* | *RecN* | *100%* |
| *B. Mallei* | *B. Pseudomallei* | *MutY* | *99.7%* |
| *B. Mallei* | *B. Pseudomallei* | *MutM* | *98.6%* |
| *B. Mallei* | *B. Pseudomallei* | *Ung* | *98.1%* |
| *MTBC* | *M. marinum* | *RecN* | *80%* |
| *MTBC* | *M. marinum* | *DNA repair exonuclease* | *Not found* |
| *MTBC* | *M. marinum* | *RecO* | *92%* |
| *MTBC* | *M. marinum* | *SbcD* | *85%* |
| *MTBC* | *M. marinum* | *Mfd* | *85%* |
| *MTBC* | *M. marinum* | *radA* | *79%* |
| *MTBC* | *M. marinum* | *MutY* | *80%* |
| *MTBC* | *M. marinum* | *Ung* | *86%* |
| *S. typhi* | *S. typhimurium* | *Mfd* | *99.7%* |
| *S. typhi* | *S. typhimurium* | *Nucleotide excision repair endonuclease* | *99.7%* |
| *S. typhi* | *S. typhimurium* | *AlkB* | *99.1%* |
| *S. typhi* | *S. typhimurium* | *RecO* | *100%* |
| *S. typhi* | *S. typhimurium* | *RecN* | *99.6%* |
| *S. typhi* | *S. typhimurium* | *MutS* | *100%* |
| *S. typhi* | *S. typhimurium* | *MutH* | *99.6%* |
| *S. typhi* | *S. typhimurium* | *RadC* | *100%* |
| *S. typhi* | *S. typhimurium* | *MutL* | *99%* |
| *S. typhi* | *S. typhimurium* | *YtfE* | *99.1%* |
| *S. typhi* | *S. typhimurium* | *RadA* | *99.6%* |
| *S. typhi* | *S. typhimurium* | *MutY* | *99.4%* |
| *S. typhi* | *S. typhimurium* | *MutM* | *99.6%* |
| *S. typhi* | *S. typhimurium* | *Ung* | *100%* |
| *S. typhi* | *S. typhimurium* | *Vsr* | *99.4%* |
| *B. anthracis* | *B. thuringiensis* | *Mfd* | *99.8%* |
| *B. anthracis* | *B. thuringiensis* | RadA | 99.8% |
| *B. anthracis* | *B. thuringiensis* | DNA repair exonuclease | 96.9% |
| *B. anthracis* | *B. thuringiensis* | MutS family protein | 99.5% |
| *B. anthracis* | *B. thuringiensis* | MutL | 95.2% |
| *B. anthracis* | *B. thuringiensis* | MutS | 99% |
| *B. anthracis* | *B. thuringiensis* | RecN | 99.3% |
| *B. anthracis* | *B. thuringiensis* | RecO | 100% |
| *B. anthracis* | *B. thuringiensis* | RadC | 100% |
| *B. anthracis* | *B. thuringiensis* | MutY | 99.5% |
| *B. anthracis* | *B. thuringiensis* | MutM | 100% |
| *B. anthracis* | *B. thuringiensis* | Ung | 97.8% |
