# Supplementary material for: Evidence That Mutation Is Universally Biased towards AT in Bacteria
Source: PLoS Genet. 2010 Sep 9;6(9):e1001115. doi: 10.1371/journal.pgen.1001115 (PMC2936535; doi:10.1371/journal.pgen.1001115)
Supplement: Table S2 — Summary of results for five low-diversity sub-clades, with singletons removed. (0.03 MB DOC) [file pgen.1001115.s003.doc]

Table S2. Summary of results for five low-diversity sub-clades, with singletons removed

| *Organism* | *Current GC intergenic* | *GC_eq_ intergenic^a^* | *Current GC non-synonymous* | *GC_eq_ non-synonymous^a^* | *Current GC synonymous* | *GC_eq_ synonymous^a^* |
| --- | --- | --- | --- | --- | --- | --- |
| *Bacillus anthracis* | 32.5 | 32.9  (24.3, 42.9) | 39.5 | **25.2**  (19.1, 31.8) | 23.4 | 30.3  (21,2, 41.6) |
| *Salmonella typhi* | 46.1 | **21.7**  (17.2,26.9) | 50.1 | **22.7**  (19.9, 25.4) | 62.3 | **18.6**  (14.9, 22.4) |
| *Yersinia pestis* | 42.5 | **23.3**  (8.5, 40.6) | 48.1 | **22.1**  (11.4, 34.9) | 51.7 | **31.9**  (12.7, 51.7) |
| *Burkholderia mallei* | 67.8 | 59.6  (31.9, 79.1) | 61.1 | **8.7**  (0, 21.3) | 90.5 | **29.4**  (0,59) |
| MTBC | 62.8 | Missing data | 60.8 | **32.8**  (21.4, 44.7) | 80.2 | **39.8**  (15.3, 58.5) |

^a^95% Confidence intervals appear in parenthesis. Bold font indicates GC_eq_ values are significantly different from corresponding current GC contents
