# Supplementary material for: Evidence That Mutation Is Universally Biased towards AT in Bacteria
Source: PLoS Genet. 2010 Sep 9;6(9):e1001115. doi: 10.1371/journal.pgen.1001115 (PMC2936535; doi:10.1371/journal.pgen.1001115)
Supplement: Table S4 — Strength of selection or selection like processes for GC over AT nucleotides assuming constant selection and no competing selective pressures. (0.04 MB DOC) [file pgen.1001115.s005.doc]

**Table S4.** Strength of selection or selection like processes for GC over AT nucleotides assuming constant selection and no competing selective pressures

| *Organism* | *sites* | *4 N_e_s* |
| --- | --- | --- |
| MTBC | Synonymous | 1.74 |
| MTBC | Non- synonymous | 0.78 |
| B. mallei | Synonymous | 3.06 |
| B. mallei | Non-synonymous | 2.29 |
| B. mallei | Intergenic | 0.81 |
| Y. pestis | Synonymous | 1.18 |
| Y. pestis | Non-synonymous | 1.11 |
| Y. pestis | Intergenic | 0.65 |
| B. anthracis | Synonymous | -0.43 |
| B. anthracis | Non-synonymous | 0.68 |
| B. anthracis | Intergenic | -0.07 |
| S. typhi | Synonymous | 1.93 |
| S. typhi | Non-synonymous | 1.17 |
| S. typhi | Intergenic | 1.07 |
